# Limited duration of dexamethasone in newly diagnosed AL amyloidosis: impact on response, toxicity, and survival

**DOI:** 10.1080/07853890.2026.2693452

**Published:** 2026-08-07

**Authors:** John Hanna, Hong Li, Ryan Brown, Zain Ali Burney, Kawther Abdallah, Diana Basali, Jason Valent, Christy Samaras, Faiz Anwer, Jack Khouri, Shahzad Raza, Willem Van Heeckeren, Mazen Hanna, J. Emanuel Finet, Louis Williams, Sandy Mazzoni

**Affiliations:** a Department of Internal Medicine, Cleveland Clinic, Cleveland, OH, USA; b Department of Quantitative Health Sciences, Cleveland Clinic Taussig Cancer Institute, Cleveland, OH, USA; c Department of Hematology and Medical Oncology, Taussig Cancer Institute, Cleveland Clinic, Cleveland, OH, USA; d Department of Cardiovascular Medicine, Heart, Vascular, and Thoracic Institute; Cleveland Clinic, Cleveland, OH, USA

**Keywords:** Light chain amyloidosis, corticosteroids, toxicity, hematologic response, organ response, treatment duration

## Abstract

**Background:**

Systemic light chain amyloidosis (AL) is a life-threatening disease in which dexamethasone (Dex) is a core therapy but is limited by cumulative toxicity. The optimal duration of Dex, particularly in the era of daratumumab (Dara)-based regimens, is uncertain.

**Methods:**

We retrospectively analyzed 216 newly diagnosed AL amyloidosis patients (2017–2023). Dex exposure was categorized as limited (≤3 months) or prolonged (>3 months) using restricted cubic spline-derived associations with hematologic response kinetics. Outcomes included hematologic and organ response, toxicity, and overall survival.

**Results:**

Median Dex duration was 5.5 months and was similar by Dara use. Overall response rates exceeded 90% at 6 months in both Dex groups. Limited Dex was associated with higher rates of early deep hematologic response (VGPR or better 77.5 vs 56.5%, *p* = 0.001; CR 42.2 vs 19.4%, *p* < 0.001), reflecting faster response kinetics, while overall response rates were similar. Among patients receiving limited Dex, Dara was associated with higher 6-month CR rates (66.7 vs 30.4%, *p* < 0.001) and increased likelihood of achieving CR in multivariable analysis (HR 4.38, 95% CI 2.44-7.85; *p* < 0.001). Organ responses were similar between groups. Dex-related toxicities increased after 6 months, and hospitalization was associated with worse survival.

**Conclusions:**

Limiting Dex exposure was associated with preserved efficacy and reduced toxicity. Prolonged Dex was associated with increased toxicity without improving overall hematologic or organ outcomes, supporting limited Dex duration in frontline AL amyloidosis therapy.

## Introduction

Systemic immunoglobulin light-chain (AL) amyloidosis is a complex and life-threatening disorder caused by a clonal plasma cell population that produces unstable immunoglobulin light chains, which misfold and deposit extracellularly as amyloid fibrils in tissues, leading to progressive organ dysfunction. The disease commonly involves multiple organs, including the heart, kidneys, gastrointestinal tract, liver, and peripheral nervous system. Cardiac involvement, leading to restrictive cardiomyopathy, is a critical determinant of prognosis and survival, with high mortality rates seen in patients with advanced heart failure [1,2]. In cardiac amyloidosis, amyloid fibrils are deposited in the myocardial interstitium, disrupting myocyte vitality and viability and impairing diastolic function and commonly disrupting the electrical system of the heart leading to dysrhythmias. This can lead to frequent hospitalizations due to acute decompensated heart failure, with prolonged lengths of stay and increased rates of in-hospital death [1,2]. Renal involvement is also common in AL amyloidosis with deposition in renal glomeruli and tubules, leading to proteinuria that can progress to renal failure with hemodialysis dependence. Hepatic involvement in AL amyloidosis involves extracellular fibril deposition leading to damage manifesting as elevated alkaline phosphatase and, in severe cases, portal hypertensive pathology. Early identification of AL amyloidosis and timely initiation of anti-plasma cell therapy is key to limiting hematologic progression, amyloid deposition, and organ damage.

The mainstay of treatment for AL amyloidosis involves reducing light chain production through anti-plasma cell chemo-immuno-therapeutic agents in combination with dexamethasone (Dex), a corticosteroid known for its immunosuppressive and anti-inflammatory effects [3]. Based on the results of the landmark ANDROMEDA study published in June 2021, Daratumumab (Dara) was added to the standard of care regimen of Bortezomib + Cyclophosphamide + Dex (CyBorD). The addition of Dara improved the rate of hematologic complete response (hCR) and both renal and cardiac organ responses [4]. Dex, when used in combination with Bortezomib or Cyclophosphamide, has shown efficacy in inducing hematologic responses by reducing light chain concentrations [5]. However, the extended use of Dex is associated with a range of severe adverse effects, including cardiovascular complications. Cardiovascular toxicity can manifest as systemic hypertension, left ventricular dysfunction, arrhythmias, fluid retention, and vascular remodeling due to glucocorticoid-induced reactive oxygen species [6,7]. These effects are particularly concerning in patients with cardiac light chain amyloidosis, where the amyloid deposits already compromise myocardial structure and function. Steroid-induced hypertension and fluid retention can exacerbate diastolic dysfunction, leading to decompensated heart failure. The increased myocardial workload and susceptibility to arrhythmias further compound the risk of adverse cardiac events. Steroids increase the demand for diuretics to manage fluid overload, which can lead to challenges in maintaining optimal volume status, frequent medication adjustments, renal impairment, and potential electrolyte disturbances. This delicate balance often results in recurrent hospitalizations for heart failure management. Additionally, hyperglycemia is frequently seen with steroid use due to upregulation of gluconeogenesis-specific enzymes, resulting in increased glucose production and insulin resistance [8]. This metabolic derangement not only adds to the cardiovascular strain but also increases the risk of infection, further contributing to the clinical instability of these patients [9]. The cumulative impact of these factors underscores how prolonged exposure to corticosteroids can cause morbidity with an increased need for medical interventions, and negatively affect the quality of life in AL patients.

Advancements in the understanding of AL amyloidosis pathophysiology have underscored the importance of early, rapid hematologic responses to prevent organ deterioration, particularly in patients with cardiac involvement. Aggressive early treatment to suppress clonal plasma cells has been shown to improve survival outcomes [2]. However, the optimal duration of Dex exposure required to achieve these responses remains uncertain, raising concerns about balancing therapeutic efficacy with the potential for significant steroid-related side effects. Given the well-documented role of Dex in contributing to early cardiovascular mortality among AL amyloidosis patients with cardiac involvement [10], strategies to reduce cumulative steroid exposure have gained attention. Sequential treatment protocols that delay or modify steroid use have been proposed to minimize cardiac-related adverse events [11]. This concept is also supported by the broader plasma cell literature. In multiple myeloma, earlier dose-reduction strategies demonstrate that reducing steroid exposure is associated with better short-term overall survival and lower toxicity compared to prolonged steroid exposure [12,13].

Reducing Dex exposure offers a promising approach to limit adverse outcomes, particularly in high-risk patients with pre-existing cardiovascular disease or impaired renal function. This highlights the need for studies evaluating whether limited Dex regimens can maintain therapeutic benefits while mitigating the risks in AL amyloidosis.

## Methods

We conducted a retrospective consecutive cohort study at the Cleveland Clinic between January 1, 2017, and January 1, 2023, including all adults with newly diagnosed systemic AL amyloidosis treated during this period. This study was conducted in accordance with the Declaration of Helsinki. The study protocol was reviewed and approved by the Cleveland Clinic Institutional Review Board (IRB) with a waiver of informed consent due to the retrospective nature of the study and minimal risk to participants (IRB 24-166). Diagnosis was confirmed by tissue biopsy demonstrating Congo red–positive amyloid deposition together with clinical and laboratory findings consistent with AL amyloidosis. Patients meeting International Myeloma Working Group (IMWG) criteria for multiple myeloma were excluded. Baseline data included demographics, clinical presentation, and organ involvement, which was defined according to International Society of Amyloidosis (ISA) criteria and assessed using N-terminal pro-B-type natriuretic peptide (NT-proBNP), 24-hour urine protein, and alkaline phosphatase (ALP) [14]. Laboratory evaluation of monoclonal gammopathy included serum M-protein, involved and uninvolved serum free-light chains (FLC), and the difference between involved and uninvolved FLC (dFLC). Hematologic involvement was assessed using serum and urine immunofixation and FLC quantification [2].

Patients received varying durations of Dex in combination with bortezomib and cyclophosphamide, with Dara incorporated into frontline regimens after January 15, 2021, following FDA approval of the ANDROMEDA regimen [4]. Prior to the incorporation of daratumumab, the majority of patients were treated with the CyBorD regimen (bortezomib, cyclophosphamide, and dexamethasone), which represented the institutional standard of care during the earlier study period. Dex duration was not protocolized and was determined by the treating clinician based on individual patient factors, including treatment tolerance, patient frailty, and adverse effects related to corticosteroid exposure. The primary endpoints were hematologic and organ responses within 24 months of Dex initiation, defined according to ISA criteria [2]. Hematologic responses included complete response (CR), very good partial response (VGPR), partial response (PR), no response, and progression. CR required a normal FLC ratio and negative serum and urine immunofixation. VGPR was defined as a reduction in dFLC to <40 mg/L. PR required *a* > 50% reduction in dFLC. Progression was defined as recurrence of an abnormal FLC ratio after CR or *a* ≥ 50% increase in serum M-protein to >0.5 g/dL or urine M-protein to >200 mg/day after any response.

Organ responses followed ISA criteria [2,14]: cardiac response required *a* ≥ 30% and ≥300 ng/L decrease in NT-proBNP (if baseline ≥650 ng/L) without *a* ≥ 30% increase; renal response required *a* ≥ 30% decrease in 24-hour proteinuria or reduction to <0.5 g/day without *a* ≥ 25% decline in estimated glomerular filtration rate; hepatic response required *a* ≥ 50% reduction in ALP in the absence of progression elsewhere.

Steroid-associated toxicities were identified through systematic review of electronic medical records and were defined as clinical events occurring during steroid exposure, including all-cause hospitalization, hospitalization for heart failure exacerbation, arrhythmias, severe hyperglycemia (grade ≥2), hypertension, fluid retention, escalation of diuretic dose, obesity developing after therapy, psychosis, and diabetes-related complications. Events were graded using Common Terminology Criteria for Adverse Events (CTCAE) version 5.0.

Patient demographics and clinical characteristics were summarized descriptively and compared in Dara vs no Dara groups using Chi square test and Wilcoxon two sample test. Dex time was compared between Dara groups using Wilcoxon two-sample test.

Primary outcomes were hematologic responses within 24 months of Dex initiation, including overall response rate (ORR; PR or better), time to first response, and time to best response. Patients who responded after 24 months or were followed beyond 24 months were censored at 24 months.

Associations between Dex duration and hematologic response were initially explored using restricted cubic spline plots. Based on these analyses, 3 months was identified as the inflection point beyond which additional Dex exposure was not associated with further improvement in response probability, and Dex exposure was therefore categorized into Limited (≤3 months) and Prolonged (>3 months) groups. In addition, a significant interaction was observed between Dex duration and Dara use; hematologic response analyses were performed across four prespecified Dex-Dara groups: Dex ≤3 months with Dara, Dex ≤3 months without Dara, Dex >3 months with Dara, and Dex >3 months without Dara. ORR, VGPR or better, and CR at 6-month and 12-month were compared between Dex ≤ 3 and > 3 months and then between Dara and no Dara at Dex ≤ 3 and > 3 months using Chi square test. Time from treatment initiation to first response of PR or better was compared among Dex-Dara groups using Kaplan-Meier method. Based on results from these analyses, formal statistical analyses focused on time to CR. Associations between Dex-Dara groups, demographic characteristics, and baseline clinical features with time to CR were assessed using Kaplan-Meier methods and Cox proportional hazards models. Variables with *p* < 0.25 in univariate analyses, as well as age, were further evaluated in multivariate analysis. A stepwise selection procedure in Cox hazard model was performed to identify factors that were independently associated with CR.

Associations between Dex duration and toxicity risk were evaluated using generalized additive models (GAM) with penalized smoothing splines to identify inflection points in risk. Based on these curves, toxicities were compared between ≤6 and >6 months of Dex exposure using Chi-square or Fisher’s exact tests where appropriate. Two-sided *p*-values <0.05 were considered statistically significant. All analyses were performed using SAS version 9.4.

## Results

### Baseline characteristics and time of Dex use

A total of 216 patients with newly diagnosed AL amyloidosis initiated dexamethasone-based therapy at the Cleveland Clinic between January 2017 and January 2023. Baseline characteristics are summarized in Table 1. Baseline characteristics stratified by dexamethasone exposure (≤3 months vs >3 months) are provided in Supplementary Table 1. The median age at treatment initiation was 66 years (IQR 59–73), with 54.2% aged >65 years. Most patients were male (65.7%) and White (81.5%), and lambda light-chain disease was present in 71.8%.

**Table 1.** Baseline demographic and clinical characteristics of AL amyloidosis patients at time of diagnosis.

Characteristic	*N* = 216
Age at treatment start (years)	66.0 (59.0, 73.0)
*Age > 65*	117 (54.2%)
Gender	
*Male*	142 (65.7%)
*Female*	74 (34.3%)
Race	
*White*	176 (81.5%)
*Black*	40 (18.5%)
Light chain type	
*Lambda*	155 (71.8%)
*Kappa*	61 (28.2%)
Baseline dFLC (mg/L)	201.5 (81.0, 620.8)
*dFLC > 180 mg/L*	113 (52.3%)
NT-proBNP (ng/L)	2235.0 (614.0, 7080.0)
*NT-proBNP > 1800 ng/L*	112 (54.6%)
24-hour urine protein (mg)	1270.0 (170.0, 4480.0)
*24-hour urine protein ≥ 150 mg*	136 (76.8%)
Alkaline phosphatase (U/L)	90.0 (70.0, 133.0)
*ALP > 130 U/L*	59 (28.0%)
Received daratumumab	60 (27.8%)
Autologous stem cell transplant	40 (18.5%)
Baseline organ involvement	
*Cardiac*	125 (66.8%)
*Renal*	108 (60.7%)
*Hepatic*	11 (7.9%)
Dara use	60 (27.8%)

Baseline demographic, laboratory, and clinical characteristics of 216 patients with newly diagnosed light-chain amyloidosis at the time of diagnosis. Continuous variables are presented as median with interquartile range, and categorical variables are presented as number and percentage.

Values are median (Q1, Q3) or *n* (%).

Abbreviations: dFLC: difference in free light chains; ALP: alkaline phosphatase; NT-proBNP: N-terminal pro–B-type natriuretic peptide.

The median baseline dFLC was 201.5 mg/L (IQR 81.0–620.8), and 52.3% had a dFLC >180 mg/L. Median NT-proBNP was 2235.0 ng/L (IQR 614.0–7080.0), with 54.6% >1,800 ng/L. Median 24-hour urine protein was 1270.0 mg (IQR 170.0–4480.0), with 76.8% ≥150 mg/24 h. Median ALP was 90.0 U/L (IQR 70.0–133.0), and 28.0% had ALP >130 U/L. Dara was incorporated into frontline therapy in 27.8% of patients, and 18.5% underwent autologous stem cell transplantation during or after frontline therapy. At baseline, 66.8% had cardiac involvement, 60.7% had renal involvement, and 7.9% had hepatic involvement.

Among 216 patients, 60 (27.8%) used Dara. Patients who did not receive daratumumab were primarily treated with the CyBorD regimen, reflecting treatment practices prior to the routine incorporation of daratumumab into frontline therapy. Patient demographics and baseline clinical characteristics were comparable between Dara and no Dara groups, except for NT-proBNP, in which 42.9% of patients in the Dara group and 59.1% in the no Dara group had an NT-proBNP > 1,800 ng/L. The median (IQR) duration of Dex exposure was 5.5 (3.9–13.5) months (range 0.39–79.1). Time of Dex use [(Median (IQR) months] was similar between Dara [5.5 (4.3, 11.4)] and no Dara [5.5 (3.8, 14.3)] groups.

### Hematologic response

**Figure 1.**
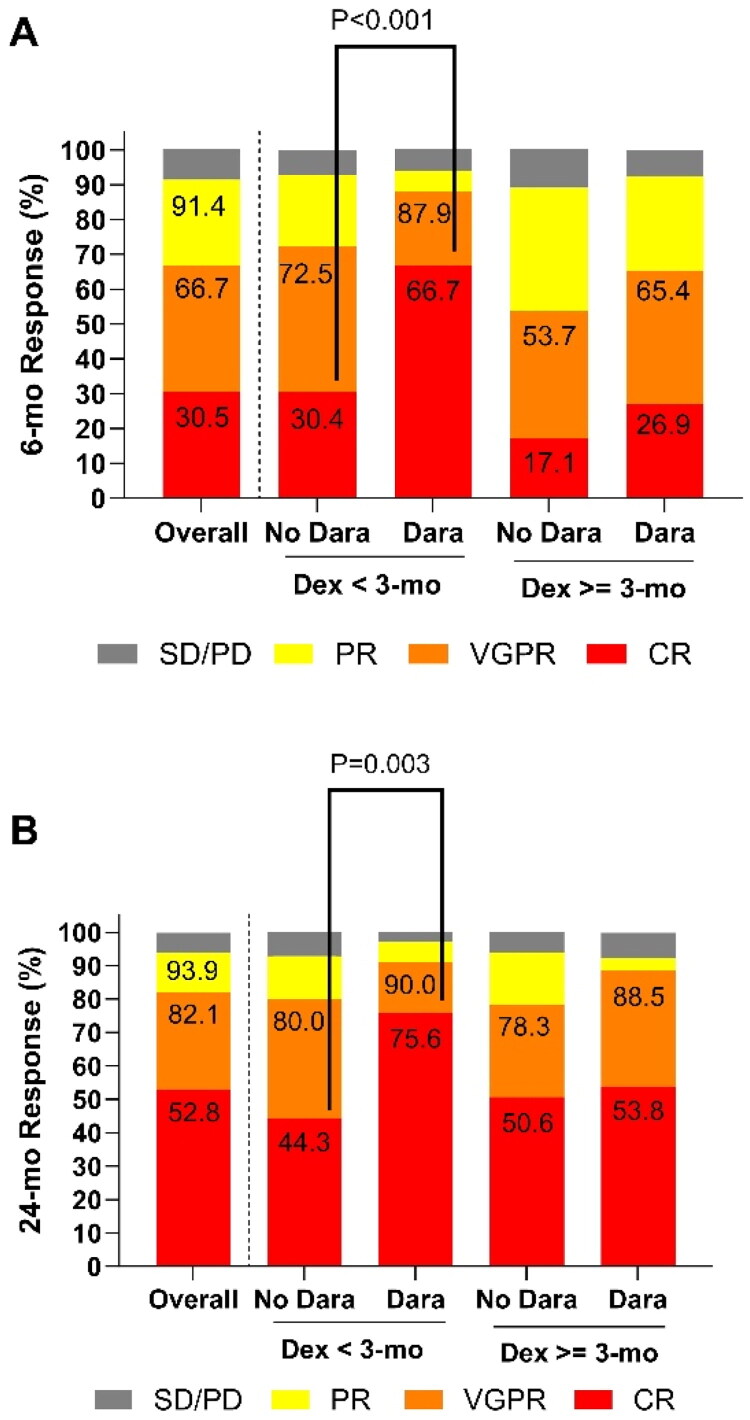
Stacked bar chart showing distribution of hematologic responses at (A) 6 months and (B) 24 months, stratified by Dexamethasone duration (≤3 months vs >3 months) and Daratumumab use. Each bar represents cumulative response rates including Complete Response (CR, red), Very Good Partial Response (VGPR, orange), Partial Response (PR, yellow), and Stable or Progressive Disease (SD/PD, grey). Abbreviations: CR, complete response; VGPR, very good partial response; PR, partial response; SD, stable disease; PD, progressive disease; Dex, dexamethasone; Dara, daratumumab.

Hematologic responses stratified by Dex duration group, are shown in Figure 1A,B. At 6 months, the ORR was 91.4% (192/216), with similar rates between Limited and Prolonged Dex groups (93.1% vs 89.8%, *p* = 0.39). However, deeper responses strongly favored Limited Dex: ≥VGPR occurred in 77.5% versus 56.5% (*p* = 0.001), and CR in 42.2% versus 19.4% (*p* < 0.001). Within the Limited Dex group, Dara produced a marked increase in CR (66.7% vs 30.4%, *p* < 0.001), whereas ≥ VGPR showed a non-significant trend favoring Dara (87.9% vs 72.5%, *p* = 0.08). In contrast, among patients receiving Prolonged Dex, Dara did not significantly affect ≥ VGPR (65.4 vs 53.7%, *p* = 0.29) or CR rates (26.9 vs 17.1%, *p* = 0.27), indicating that extending Dex beyond 3 months did not further improve response depth.

When examining time to first ORR, results were consistent, with no significant differences between Dara and no Dara patients within either the Limited Dex group [median months (95% CI): 1.02 (0.7–1.8) vs 1.1 (0.9–1.8)] for the Prolonged Dex group [2.7 (1.5–3.9) vs 2.7 (1.8–3.6)].

To further evaluate response kinetics, time to CR and factors associated with CR were analyzed using Kaplan-Meier estimates and Cox regression (Table 2). Because this analysis evaluates time to complete response, CR hazard ratios (CR HR) represent the relative likelihood of achieving CR over time (response kinetics), rather than differences in final CR proportions between treatment groups. A significant interaction between Dex duration and Dara use on time to CR was identified (*p* < 0.001), and time to CR was therefore evaluated across four Dex-Dara categories.

**Table 2.** Association of dexamethasone–daratumumab exposure with time to complete hematologic response.

Variable	*N*	CR events (%)	Median time to CR (months)	6-month CR % (95% CI)	Cox univariate HR (95% CI)	*P* value	Adjusted HR for CR (95% CI)	*P* value
Dex–Dara groups								
Dex ≤3 months/No Dara	70	31 (44%)	4.1	51.6 (35.0, 68.2)	Reference		Reference	
Dex ≤3 months/Dara	33	25 (76%)	1.6	86.5 (71.2, 100)	4.27 (2.42, 7.51)	<0.001	4.38 (2.44, 7.85)	<0.001
Dex >3 months/No Dara	83	42 (51%)	12.5	14.6 (6.6, 22.6)	0.52 (0.32, 0.84)	0.008	0.51 (0.31, 0.83)	0.007
Dex >3 months/Dara	26	14 (54%)	11.7	28.7 (10.7, 46.7)	0.52 (0.28, 0.99)	0.045	0.45 (0.23, 0.87)	0.017
Pairwise comparisons								
1 vs 3					1.91 (1.18, 3.08)	0.008	1.97 (1.20, 3.23)	0.007
2 vs 4					8.19 (4.09, 16.38)	<0.001	9.72 (4.68, 20.20)	<0.001
3 vs 4					1.00 (0.55, 1.84)	0.99	1.13 (0.60, 2.10)	0.71
Age (years)								
≤65	98	56 (57%)	8	33.9 (23.9, 44.0)	1.13 (0.78, 1.64)	0.52	1.61 (1.05, 2.47)	0.03
>65	114	56 (49%)	8	40.1 (29.6, 50.6)	Reference		Reference	
Gender								
Female	72	40 (56%)	7.3	39.6 (27.3, 51.9)	Reference			
Male	140	72 (51%)	8.4	35.6 (26.5, 44.6)	0.96 (0.65, 1.41)	0.83		
Race								
Non-Black	174	97 (56%)	7.4	40.5 (32.2, 48.7)	Reference			
Black	38	15 (39%)	12.5	21.4 (7.2, 35.7)	0.58 (0.34, 0.99)	0.049		
Light chain type								
Kappa	59	24 (41%)	14.7	29.5 (16.1, 43.0)	Reference			
Lambda	153	88 (58%)	7.6	39.5 (31.0, 48.1)	1.42 (0.90, 2.23)	0.13		
dFLC (mg/L)								
≤180	103	59 (57%)	6.9	44.2 (33.5, 55.0)	1.60 (1.10, 2.33)	0.013	1.56 (1.06, 2.31)	0.03
>180	109	53 (49%)	9.3	29.9 (20.3, 39.6)	Reference		Reference	
NT-proBNP (ng/L)								
≤1800	93	49 (53%)	7.6	37.0 (26.2, 47.8)	Reference			
>1800	109	57 (52%)	8.7	36.5 (26.3, 46.7)	0.96 (0.66, 1.41)	0.85		
24-h urine protein ≥150 mg								
<150	41	23 (56%)	7.3	27.6 (12.7, 42.6)	Reference			
≥150	133	69 (52%)	8.6	40.6 (31.2, 50.0)	0.98 (0.61, 1.58)	0.94		
ALP (U/L)								
≤130	149	76 (51%)	10.9	32.3 (23.9, 40.6)	Reference		Reference	
>130	58	34 (59%)	4.9	50.8 (36.1, 65.6)	1.81 (1.20, 2.74)	0.005	1.64 (1.08, 2.50)	0.02
Cardiac transplantation								
No	199	100 (50%)	8.7	65.3 (57.9, 72.8)	Reference			
Yes	13	12 (92%)	4.8	35.2 (8.0, 62.3)	2.21 (1.21, 4.03)	0.01		
Autologous stem cell transplantation								
No	172	89 (52%)	7.4	38.2 (30.0, 46.4)	1.33 (0.84, 2.11)	0.23	1.80 (1.05, 3.07)	0.03
Yes	40	23 (58%)	14.1	31.7 (15.9, 47.5)	Reference		Reference	

Kaplan–Meier estimates and cox proportional hazards models evaluating time to complete hematologic response according to dexamethasone duration and daratumumab exposure. Dexamethasone exposure was categorized as ≤3 months or >3 months, with or without daratumumab. A significant interaction between dexamethasone duration and daratumumab use was observed (P for interaction <0.001); therefore, analyses were performed across four Dex–Dara groups. Hazard ratios (CR HR) reflect the relative likelihood of achieving complete response over time (response kinetics), rather than differences in final CR proportions; values >1 indicate faster achievement of CR.

Abbreviations: CI, confidence interval; CR, complete response; Dara, daratumumab; Dex, dexamethasone; HR, hazard ratio; dFLC, difference between involved and uninvolved free light chains; NT-proBNP, N-terminal pro–B-type natriuretic peptide; ALP, alkaline phosphatase.

At 6 months, CR rates were highest among patients receiving limited Dex with Dara (86.5%), followed by limited Dex without Dara (51.6%), whereas substantially lower CR rates were observed in the prolonged Dex groups (28.7% with Dara and 14.6% without Dara), consistent with slower response kinetics.

In univariate analysis, Dara markedly accelerated time to CR among patients receiving ≤3 months of Dex, with a median time to CR of 1.6 vs 4.1 months and higher 6-month CR rates (86.5 vs 51.6%). In contrast, among patients treated with >3 months of Dex, CR kinetics were similar regardless of Dara exposure (median 11.7 vs 12.5 months; 6-month CR rates remained low and similar (28.7 vs 14.6%). Although the crude CR proportion was numerically higher in the prolonged Dex/no-Dara group compared with the limited Dex/no-Dara group, responses occurred substantially later, indicating slower response kinetics rather than differences in final response rates. Consistent with this, pairwise comparisons demonstrated significantly faster time to CR with limited Dex compared with prolonged Dex both in the absence of daratumumab (adjusted HR 1.97, *p* = 0.007) and, more prominently, in the presence of daratumumab (adjusted HR 9.72, *p* < 0.001). Organ involvement at diagnosis was not associated with CR. In multivariate analysis, Dex-Dara group remained significant. Within the ≤3-month Dex group, Dara resulted in more than a fourfold higher likelihood of achieving CR (HR 4.38, 95% CI 2.44–7.85; *p* < 0.001), whereas no difference was observed between Dara and no Dara among patients receiving >3 months of Dex. Additional independent predictors of earlier CR included age <65 years (HR 1.61, 95% CI 1.05–2.47; *p* = 0.03), baseline dFLC ≤180 mg/L (HR 1.56, 95% CI 1.06–2.31; *p* = 0.03), ALP >130 U/L (HR 1.64, 95% CI 1.08–2.50; *p* = 0.02), and no autologous stem cell transplantation (HR 1.80, 95% CI 1.05–3.07; *p* = 0.03).

At 24 months, ORR remained comparable between Limited and Prolonged Dex (94.2 vs 93.6%, *p* = 0.86), and rates of ≥ VGPR (83.5 vs 80.7%, *p* = 0.60) and CR (54.4 vs 51.4%, *p* = 0.66) were also similar. The benefit of Dara persisted within the Limited Dex cohort, with CR rates of 75.8 vs 44.3% (*p* = 0.003), whereas CR rates in the Prolonged Dex group remained similar regardless of Dara exposure (53.8 vs 50.6%, *p* = 0.77).

### Organ response

Organ response assessments at 6, 12, 18, and 24 months were available for 125 patients with cardiac involvement, 108 with renal involvement, and 11 with hepatic involvement. Across these patients, no significant differences in cardiac, renal, or hepatic response rates were observed between the Limited (≤3 months) and Prolonged (>3 months) Dex groups at any time point. Similarly, when Dex duration was categorized as ≤6 versus >6 months, organ response rates remained comparable, indicating that a shorter Dex course did not compromise cardiac, renal, or hepatic recovery. These findings suggest that while hematologic responses differed by Dex exposure, organ responses appeared unaffected by the duration of steroid therapy.

### Steroid-associated toxicities

**Figure 2.**
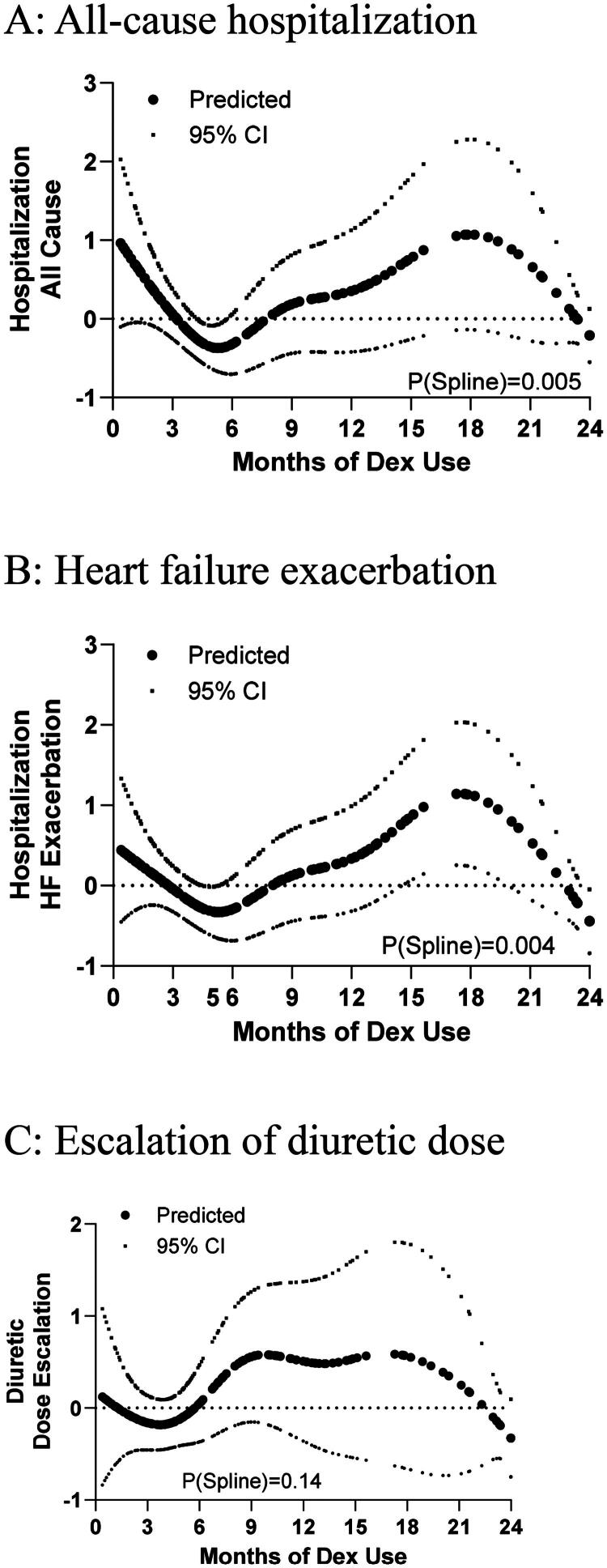
Dexamethasone (Dex) exposure and toxicity risk within 24 months. (A) All-cause hospitalization, (B) Hospitalization for heart failure exacerbation, and (C) Escalation of diuretic dose. Penalized smoothing splines display a notable inflection at 6 months of Dex exposure, after which the probability of each toxicity increases substantially.

Associations between Dex duration and steroid-related toxicities are shown in Table 3 and Figure 2A–C. The relative risk of all-cause hospitalization (Figure 2A), hospitalization for heart-failure exacerbation (Figure 2B), and escalation of diuretic therapy (Figure 2C) increased progressively with longer Dex exposure, with a noticeable rise beginning beyond approximately 6 months. When Dex duration was dichotomized at this 6-month inflection point, patients receiving >6 months of Dex had higher rates of any hospitalization (75.5 vs 60.7%, *p* = 0.02), heart-failure exacerbation (43.4 vs 29.1%, *p* = 0.03), and diuretic escalation (82.8 vs 62.4%, *p* < 0.001) compared with those treated for ≤6 months (Table 3). Arrhythmias (*p* = 0.06) and severe hyperglycemia (*p* = 0.06) showed trends toward higher frequency with prolonged Dex exposure, whereas hypertension, fluid retention, and obesity were not significantly different between the two Dex groups. Psychosis was observed exclusively among patients receiving >6 months of Dex (4.0 vs 0%, *p* = 0.04). Collectively, these findings highlight the association between longer steroid exposure and increased toxicity and support limiting Dex duration when clinically feasible.

**Table 3.** Steroid-associated toxicities in patients treated with dexamethasone ≤6 months vs >6 months.

Toxicity	Dex ≤ 6 months (*N* = 117)	Dex > 6 months (*N* = 99)	Total (*N* = 216)	*p*-value
Any hospitalization	71 (60.7%)	74 (75.5%)	145 (67.4%)	0.02
HF-related hospitalization	34 (29.1%)	43 (43.4%)	77 (35.6%)	0.03
Arrhythmias	13 (11.1%)	20 (20.2%)	33 (15.3%)	0.06
Severe hyperglycemia (grade ≥ 2)	21 (17.9%)	9 (9.1%)	30 (13.9%)	0.06
Escalation of diuretics	73 (62.4%)	82 (82.8%)	155 (71.8%)	<0.001
Hypertension (grade ≥ 2)	33 (28.7%)	29 (29.3%)	62 (29.0%)	0.92
Fluid retention (grade ≥ 2)	51 (43.6%)	46 (46.5%)	97 (44.9%)	0.67
Obesity developing post-treatment	29 (25.4%)	26 (27.4%)	55 (26.3%)	0.75
Psychosis (any grade)	0 (0%)	4 (4.0%)	4 (1.9%)	0.04
Diabetes complications	2 (1.7%)	2 (2.0%)	4 (1.9%)	0.99
Hyperglycemia (grade 3–4)	9 (7.7%)	3 (3.0%)	12 (5.6%)	0.14
Hypertension (grade 3–4)	14 (12.2%)	10 (10.1%)	24 (11.2%)	0.63
Fluid retention (grade 3–4)	23 (19.7%)	21 (21.2%)	44 (20.4%)	0.78
Psychosis (grade 3–4)	0 (0%)	3 (3.0%)	3 (1.4%)	0.10

Steroid-associated toxicities observed in patients treated with dexamethasone for ≤6 months versus >6 months. Values are presented as number and percentage.

Abbreviations: Dex, dexamethasone; HF, heart failure.

### Effect of steroid-associated toxicities on overall survival

The median (IQR) follow-up time was 3.2 (1.9–4.8) years. Early mortality was low, with 1 patient (0.5%) dying within 3 months, 7 (3.2%) within 6 months, 20 (9.3%) within 12 months, and 40 (18.5%) within 24 months. No significant association between dexamethasone duration and overall survival was observed, with similar 3-year OS between patients receiving <3 months versus ≥3 months of Dex (71.4 vs 75.4%, *p* = 0.52), although the study was not designed or powered to detect survival differences between exposure groups. The events classified in this analysis as steroid-associated toxicities—any hospitalization, heart-failure hospitalization, and escalation of diuretic therapy—were each associated with worse overall survival (Table 4). Patients who experienced any hospitalization had worse OS (3-year OS: 67.6 vs 90.2%; HR 3.72, 95% CI 1.76–7.85; *p* < 0.001). Heart-failure–related hospitalization was similarly associated with worse OS (3-year OS: 64.7 vs 80.1%; HR 1.71, 95% CI 1.02–2.85; *p* = 0.04). Escalation of diuretic therapy showed a non-significant trend toward inferior survival (72.3 vs 80.7%; HR 1.41, 95% CI 0.78–2.58; *p* = 0.26).

**Table 4.** Impact of steroid-associated toxicities on overall survival.

Variable (Reference)	*N*	Deaths *n* (%)	3-year OS % (95% CI)	Hazard ratio (95% CI)	*p*-value
Dex					
<3 months	39	12 (31%)	71.4 (56.2,86.5)		0.52
≥3 months	177	47 (27%)	75.4 (68.8,82.0)		
Any hospitalization					<0.001
No	70	8 (11%)	90.2 (82.7–97.7)	Reference	–
Yes	145	50 (34%)	67.6 (59.7–75.6)	3.72 (1.76–7.85)	<0.001
HF hospitalization					0.041
No	139	32 (23%)	80.1 (73.2–87.0)	Reference	–
Yes	77	27 (35%)	64.7 (53.4–75.9)	1.71 (1.02–2.85)	0.041
Diuretic escalation					0.26
No	61	14 (23%)	80.7 (70.4–91.0)	Reference	–
Yes	155	45 (29%)	72.3 (64.9–79.7)	1.41 (0.78–2.58)	0.26

Overall survival outcomes according to the presence of steroid-associated toxicities. Three-year overall survival estimates and hazard ratios were derived using Kaplan-Meier analysis and cox proportional hazards models, respectively. Comparisons are shown relative to the reference groups indicated.

Abbreviations: Dex, dexamethasone; HF, heart failure; OS, overall survival; CI, confidence interval; HR, hazard ratio.

## Discussion

This study evaluated the association between dexamethasone exposure duration and hematologic response, organ response, and the impact of treatment-related toxicities on overall survival in patients with newly diagnosed AL amyloidosis. We demonstrate that early discontinuation of Dex was associated with preserved overall hematologic response rates, while producing faster and deeper early hematologic responses. These benefits were most pronounced when Dara was included in the regimen, suggesting a synergistic effect between limited steroid exposure and CD38-targeted therapy. Importantly, early Dex discontinuation was associated with fewer severe toxicities, without compromising long-term organ responses. These findings support a treatment paradigm in which limited Dex exposure maintains efficacy, enhances tolerability, and leverages the therapeutic benefit of daratumumab in AL amyloidosis.

Although Dex remains central to induction regimens, its cumulative toxicities limit tolerability. In the era of Dara-based therapy, defining the optimal duration of corticosteroid exposure has become increasingly important. Because Dex duration in this cohort was clinician-determined and not protocolized, comparisons between limited and prolonged Dex exposure should be interpreted within the context of potential confounding by indication, including differences in baseline frailty, cardiac involvement, comorbidities, treatment era, and early toxicity. Although hematologic response kinetics may influence treatment decisions in clinical practice, Dex exposure in this dataset was not defined according to response status. In our cohort, hematologic responses were robust across all durations; however, patients who discontinued Dex within 3 months, particularly when combined with Dara, achieved the most rapid and deepest responses. Since dexamethasone is administered as part of combination therapy, we evaluated whether the association between dexamethasone duration and response differed according to daratumumab exposure, which demonstrated a significant Dex-Dara interaction on time to complete response. In this subgroup, responses were faster and deeper compared with those not receiving Dara. By contrast, extending Dex beyond 3 months delayed responses and did not improve organ outcomes. These results add to the growing consensus that prolonged Dex does not substantially enhance hematologic or organ responses in patients with AL amyloidosis [15]. For instance, Bianchi et al. reported that escalating steroid exposure in AL amyloidosis conferred little additional benefit and that steroid-sparing regimens can reduce treatment-related toxicity [16]. Similarly, Vaxman et al. and Basset et al. found that early hematologic control, rather than extended corticosteroid use, was the key driver of improved survival outcomes [17,18].

These findings align with and expand upon results from the ANDROMEDA trial, which suggested that the therapeutic contribution of Dex occurs primarily during the early treatment phase. In ANDROMEDA, although the starting dose was 40 mg weekly, over one-quarter of patients required dose reduction to 20 mg due to age or comorbidities, yet the Dara arm still achieved deep responses (CR 53.3%, ≥VGPR 78.5%), underscoring the potency of Dara independent of steroid intensity [4]. Similarly, in our cohort, Dex discontinuation within 3 months alongside Dara was associated with the greatest benefit, while prolonged Dex exposure added toxicity without efficacy gains. These findings suggest that most hematologic benefit occurred early in therapy and optimal tapering strategies once light-chain suppression is achieved require prospective evaluation [19]. The early discontinuation of Dex approach is further supported by our analysis of organ response, which showed no significant differences between Limited and Prolonged Dex, nor between ≤6 versus >6 months. These findings suggest that extending corticosteroid use offers no added benefit in promoting organ recovery. Rather, they support a response-adapted strategy in which Dex can be safely minimized to maintain efficacy while enhancing tolerability, especially in patients with cardiac or renal involvement who are most vulnerable to steroid-related complications [11,20].

In this study, events classified as steroid-associated toxicities were strongly associated with poorer clinical outcomes. Patients receiving longer Dex exposure experienced more frequent hospitalizations, heart-failure exacerbations, diuretic escalation, and psychiatric complications, all of which substantially affect morbidity and quality of life [21–23]. Because these events were not formally adjudicated as causally related to dexamethasone exposure, they may reflect complications of underlying disease severity or the natural clinical course of AL amyloidosis rather than direct steroid-specific effects. In addition, patients receiving longer dexamethasone exposure survived long enough to accumulate both treatment exposure and clinical events, introducing potential time-dependent bias in the interpretation of these associations. Although these toxicity-related events were associated with inferior survival, the present study was not designed to determine survival differences according to dexamethasone exposure. Instead, the overall pattern highlights the clinical relevance of minimizing prolonged steroid exposure when feasible, consistent with prior work supporting individualized, response-adapted steroid discontinuation in AL amyloidosis [17].

This study has several notable strengths. It analyzes a large, consecutive cohort of 216 newly diagnosed AL amyloidosis patients treated between 2017 and 2023, with outcomes assessed using standardized International Society of Amyloidosis criteria at prespecified time points. The exposure definition reflects real clinical decision points: Limited versus Prolonged Dex using *a* ≤ 3-month cutoff for efficacy/response kinetics, and *a* ≤ 6-month cutoff for toxicity, aligning the analysis with how steroids are tapered or continued in practice. Depth and speed of hematologic response were evaluated alongside organ responses, providing an integrated view of efficacy, and adverse events were captured in clinically meaningful terms. Importantly, the study incorporates modern therapy by explicitly modeling the significant interaction between Dex duration and Dara use and applies multivariable Cox regression to identify independent predictors of complete response, which strengthens internal validity and clinical interpretability.

While these findings align partially with historical data, certain limitations must be acknowledged. This study is retrospective and observational, so Dex duration was not randomized and may reflect confounding by indication despite multivariable adjustment. Formal Mayo cardiac staging and renal staging were not consistently recorded as categorical variables in the retrospective dataset and therefore could not be reconstructed for all patients. However, the principal biomarkers used to determine these staging systems, including NT-proBNP and 24-hour urine protein, are reported in Table 1 to characterize baseline disease severity within the cohort. Treatment practices also shifted during the study period, particularly with the incorporation of daratumumab into frontline therapy after 2021, resulting in a cohort that reflects both pre- and post-daratumumab treatment eras and which may influence outcomes across treatment periods. Additionally, cumulative dexamethasone dose and dose intensity were not consistently available in the retrospective dataset due to variable dose reductions, treatment interruptions, and individualized dosing adjustments during therapy, and therefore could not be incorporated into the present analysis. However, dexamethasone duration was highly correlated with total administered dose based on initial dose and assuming no dose adjustment or interruption (Spearman *ρ* = 0.93, *p* < 0.0001), supporting its use as a surrogate measure of steroid exposure. Such variability in steroid dosing and treatment interruptions may also have influenced response kinetics within the Prolonged Dex cohort. Future studies should incorporate cycle-level treatment data to better elucidate the impact of early therapy interruptions on response kinetics and survival. Finally, despite our study being multicenter, it is confined to one geographical area, which limits its generalizability. Prospective, response-adapted trials with biomarker-guided tapering, patient-reported outcomes, and standardized toxicity assessments are needed to validate these findings.

## Conclusions

In summary, limited dexamethasone exposure was associated with similar overall hematologic and organ responses and substantially lower treatment-related toxicity. Daratumumab remained the dominant driver of deep hematologic responses, with the greatest benefit observed when used alongside limited dexamethasone exposure, underscoring the minimal added value of prolonged steroid use in the modern treatment era. Events classified as steroid-associated toxicities, including fluid overload and heart failure exacerbations, were associated with worse overall survival. These findings support a response-adapted strategy in which dexamethasone is concentrated in the early treatment period and tapered once light chain suppression is achieved. Prospective studies should evaluate biomarker guided tapering, adjudicated toxicity assessment, and patient reported outcomes to validate steroid limiting approaches and refine frontline management of AL amyloidosis.

## Supplementary Material

Supplemental Materials AL Amyloidosis.docx

## Data Availability

The data supporting this study’s findings are available from Sandy Mazzoni upon reasonable request, at mazzons@ccf.org.
