# Supplementary material for: Limited duration of dexamethasone in newly diagnosed AL amyloidosis: impact on response, toxicity, and survival
Source: Ann Med. 2026 Aug 7;58(1):2693452. doi: 10.1080/07853890.2026.2693452 (PMC13455702; doi:10.1080/07853890.2026.2693452)
Supplement: Supplementary file 1 [file IANN_A_2693452_SM7525.docx]

**Limited Duration of Dexamethasone in Newly Diagnosed AL Amyloidosis: Impact on Response, Toxicity, and Survival**

**Supplementary Material**

John Hanna, MD¹, Hong Li, MS^2^, Ryan Brown, MD¹, Zain Ali Burney, MD¹, Kawther Abdallah, MD¹, Diana Basali, MD^3^, Jason Valent, MD^3^, Christy Samaras, DO^3^, Faiz Anwer, MD^3^, Jack Khouri, MD^3^, Shahzad Raza, MD^3^, Willem Van Heeckeren, MD, PhD^3^, Mazen Hanna, MD^4^, J. Emanuel Finet, MD^4^, Louis Williams, MD^3^, Sandy Mazzoni, DO^3^

¹Department of Internal Medicine, Cleveland Clinic, Cleveland, OH, USA. ^2^Department of Quantitative Health Sciences, Cleveland Clinic Taussig Cancer Institute, Cleveland, OH. ^3^Department of Hematology and Medical Oncology, Taussig Cancer Institute, Cleveland Clinic, Cleveland, OH, USA. ^4^Department of Cardiovascular Medicine; Heart, Vascular, and Thoracic Institute; Cleveland Clinic, Cleveland, OH, USA

**Supplementary Table 1:** **Baseline Demographic and Clinical Characteristics Stratified by Dexamethasone Exposure**

Baseline demographic, laboratory, and clinical characteristics stratified by dexamethasone exposure (≤3 months vs >3 months). Continuous variables are presented as median with interquartile range, and categorical variables are presented as number and percentage.

| **Characteristic** | **Total (N=216)** | **Dex ≤3 months (N=107)** | **Dex >3 months (N=109)** | **P value** |
| --- | --- | --- | --- | --- |
| **Age at treatment start (years)** | 66.0 (59.0, 73.0) | 67.0 (59.0, 73.0) | 65.0 (60.0, 73.0) | 0.64 |
| *Age > 65* | 117 (54.2%) | 63 (58.9%) | 54 (49.5%) | 0.17 |
| **Gender** |  |  |  |  |
| *Male* | 142 (65.7%) | 67 (62.6%) | 75 (68.8%) | 0.34 |
| **Race** |  |  |  |  |
| *Black* | 40 (18.5%) | 18 (16.8%) | 22 (20.2%) | 0.52 |
| **Light chain type** | |  |  |  |
| *Lambda* | 155 (71.8%) | 76 (71.0%) | 79 (72.5%) | 0.81 |
| **dFLC > 180 mg/L** | 113 (52.3%) | 51 (47.7%) | 62 (56.9%) | 0.18 |
| **NT-proBNP > 1800 ng/L*** | 112 (54.6%) | 58 (57.4%) | 54 (51.9%) | 0.43 |
| **24-hour urine protein ≥ 150 mg*** | 136 (76.8%) | 72 (80.0%) | 64 (73.6%) | 0.31 |
| **ALP > 130 U/L*** | 59 (28.0%) | 37 (34.9%) | 22 (21.0%) | 0.024 |
| **Cardiac transplant during/after Dex treatment** | 13 (6.0%) | 6 (5.6%) | 7 (6.4%) | 0.8 |
| **Autologous stem cell transplant during/after Dex treatment** | 40 (18.5%) | 17 (15.9%) | 23 (21.1%) | 0.32 |
| **Organ involvement at diagnosis** | | |  |  |
| *Cardiac* | 125 (66.8%) | 61 (67.8%) | 64 (66.0%) | 0.79 |
| *Renal* | 108 (60.7%) | 58 (65.9%) | 50 (55.6%) | 0.16 |
| *Hepatic* | 11 (7.9%) | 6 (9.7%) | 5 (6.5%) | 0.54 |

Values are median (Q1, Q3) or n (%). P values were calculated using the Wilcoxon rank-sum test for continuous variables and Pearson’s chi-square test or Fisher’s exact test for categorical variables, as appropriate.

**Abbreviations:** dFLC: difference in free light chains; ALP: alkaline phosphatase; NT-proBNP: N-terminal pro–B-type natriuretic peptide.
